# Efficacy and safety of leflunomide combined with corticosteroids for the treatment of IgA nephropathy: a Meta-analysis of randomized controlled trials

**DOI:** 10.1080/0886022X.2022.2085576

**Published:** 2022-07-04

**Authors:** Guangxin Lv, Chengyuan Ming

**Affiliations:** a Key Laboratory of Microecology-immune Regulatory Network and Related Diseases, School of Basic Medicine, Jiamusi University, Jiamusi, Heilongjiang Province, China; b Department of Urology, Jiamusi Central Hospital, Jiamusi, China

**Keywords:** Leflunomide, corticosteroids, IgA nephropathy, randomized controlled trials, meta-analysis

## Abstract

**Objective:**

We performed a meta-analysis of randomized controlled trials (RCTs) to evaluate the efficacy and safety of leflunomide combined with corticosteroids, compared with corticosteroids alone, for IgA nephropathy.

**Materials and methods:**

Studies were retrieved by searching of PubMed, Embase, Cochrane’s Library, China National Knowledge Infrastructure (CNKI), and Wanfang databases on 11 October 2021. A random-effect model incorporating the heterogeneity was used to pool the results. The efficacy outcomes included the complete remission rate of proteinuria, overall response rate (the combined rates of patients with complete and partial remission of proteinuria), changes of urine protein excretion (UPE), serum creatinine (SCr), and estimated glomerular infiltrating rate (eGFR).

**Results:**

Nineteen studies were included. Patients receiving the combined therapy had a higher complete remission rate (relative risk [RR]: 1.29, 95% CI: 1.08–1.55, *p* = 0.006; *I*^2^ = 0%) and overall response rate (RR: 1.18, 95% CI: 1.10–1.26, *p* < 0.001, *I*^2^ = 0%) compared to patients who received CS alone. Besides, combined therapy was associated with significantly reduced levels of UPE (mean difference [MD]: −0.30 g/24h, 95% CI: −0.43 to −0.16, *p* < 0.001; *I*^2^ = 34%) and SCr (MD: −7.55 mmol/L, 95% CI: −11.06 to −4.04, *p* < 0.001; *I*^2^ = 34%), and increased level of eGFR (MD: 6.51 mL/min/1.73 m^2^, 95% CI: 4.06–8.97, *p* < 0.001; *I*^2^ = 0%). The incidence of adverse events was not significantly different.

**Conclusions:**

Combined treatment with leflunomide and corticosteroids was more effective than corticosteroids alone for patients with IgA nephropathy.

## Introduction

IgA nephropathy (IgAN) is the most common primary glomerular disease of the global population, which has become an important cause of end-stage renal disease (ESRD) [1,2]. Pathologically, autoimmunity has been recognized as a major mechanism underlying the pathogenesis and progression of IgAN [1,3]. Accordingly, various immunosuppressants have been applied to prevent the deterioration of renal function and attenuate the proteinuria in patients with IgAN [4–8]. Conventionally, corticosteroid (CS) is a common choice for patients with IgAN [9,10]. Although previous studies have shown that CS could preserve renal function and reduce proteinuria in patients with IgAN, CS also increases the risks of several adverse events, such as gastrointestinal (GI) adverse events, infection and elevated glucose and blood pressure (BP) etc [9]. Besides, many immunosuppressants have been proposed as alternative treatments for IgAN [11]. Among them, leflunomide, an immunosuppressant functions as an inhibitor of pyridine synthesis, has been widely applied for patients with rheumatoid and kidney diseases in recent years [12,13]. An initial randomized controlled trial (RCT) showed that leflunomide was comparable to fosinopril in reducing proteinuria in IgAN [14]. In addition, combined with prednisone has also shown to be effective for the treatment of the phospholipase A2 receptor-associated primary membranous nephropathy [15]. However, subsequent RCTs comparing the efficacy and safety of the combined treatment with leflunomide and CS with CS alone showed inconsistent results [16–34]. These trials are generally of limited sample sizes [16–34], and a meta-analysis pooling the results of these trials is important for the systematical evaluation of the efficacy of the combined treatment. Accordingly, we performed a systematic review and meta-analysis to investigate the efficacy and safety of the combined treatment with leflunomide and CS for IgAN.

## Materials and methods

This systematic review and meta-analysis was performed in accordance to the PRISMA [35,36] (Preferred Reporting Items for Systematic Reviews and Meta-Analyses) statement and the Cochrane Handbook [37] guidelines. The protocol of the manuscript was registered on INPLASY (https://inplasy.com/) with the registration number INPLASY202230158.

### Search strategy

PubMed, Embase and the Cochrane Library (Cochrane Center Register of Controlled Trials), China National Knowledge Infrastructure (CNKI), and Wanfang databases were systematically searched for relevant RCTs, using the combination of the following three groups of terms: (1) ‘IgA nephropathy’ OR ‘immunoglobulin A nephropathy’ OR ‘IgA nephritis’ OR ‘IgA glomerulonephritis’ OR ‘Berger's disease’ OR ‘IgAN’; (2) ‘leflunomide’; and (3) ‘random’ OR ‘randomly’ OR ‘randomized’ OR ‘randomized’. The full search strategy for PubMed is shown in the Supplemental Online Material 1. The search was limited to studies in humans. We also analyzed reference lists of the original and review articles using a manual approach. The final database searching was performed on October 11, 2021.

### Study selection

Studies were included if they met the following criteria: (1) full-length articles published in English or Chinese; (2) reported as RCTs with parallel design; (3) included adult patients with biopsy-proved IgAN; (4) patients were randomly assigned to a treatment group of the combined therapy with leflunomide and CS, and a control group with CS alone; and (5) reported at least one of the following outcomes, including the efficacy outcomes: incidence of complete remission (CR) of the proteinuria, overall response (defined as CR or partial remission [PR] of proteinuria), changes of urine protein excretion (UPE, g/24h), serum creatinine (SCr), and estimated glomerular infiltrating rate (eGFR), and the safety outcomes including the incidence of adverse events, such as any GI discomfort, elevated alanine aminotransferase (ALT) and/or raised aspartate aminotransferase (AST), infection, elevated glucose, and elevated BP that require medical treatment. In general, CR was defined as proteinuria less than 0.15–0.3 g/day and a normal Scr level [4]. PR was defined as proteinuria reduced to at least half of the baseline measurement and an absolute value of > 0.3 g/day, as well as a relatively stable SCr level (variation less than 25%) [4]. Reviews, observational studies, crossover studies, studies including non-IgAN patients, studies including children or adolescents, and studies without available outcome data were excluded from the meta-analysis.

### Data extraction and quality assessment

Two authors independently performed the literature search, data extraction, and quality assessment according to inclusion criteria. Discrepancies were resolved by consensus. The following data was collected, such as the design characteristics, baseline characteristics of the included patients (age, gender, baseline proteinuria, SCr, eGFR, Lee classification), dosages of leflunomide and CS in the interventional and control groups, follow-up duration, concurrent use of angiotensin converting enzyme inhibitors (ACEIs) or angiotensin receptor blockers (ARBs), and definition of CR/PR outcomes. We used the seven-domain Cochrane Risk of Bias Tool [37] to evaluate the quality of the included studies, which include criteria concerning sequence generation, allocation concealment, blinding of participants and personnel, blinding of outcome assessors, incomplete outcome data, selective outcome reporting and other potential threats to validity.

### Statistical analysis

Continuous variables were analyzed using mean difference (MD), whereas dichotomous variables were analyzed using risk ratios (RR) with 95% confidence interval (CI). Cochrane’s *Q* test was applied to evaluate the heterogeneity among the included studies. The *I*^2^ statistic was also determined, which indicates the percentage of total variation across studies that is due to the heterogeneity rather than chance [37,38]. An *I*^2^ > 50% indicates significant heterogeneity among the trials. A random-effect model was used to pool the results since this model was considered to incorporate the potential between-study heterogeneity and could therefore minimize the influence of possible heterogeneity on the result [37]. Predefined subgroup analyses [37] were used to evaluate whether the results were consistent for studies with full-dose or reduced dose of CS in the combined therapy. For efficacy outcomes with at least ten datasets, univariate meta-regression analyses according to the following study characteristics were performed, including sample size, mean age, proportions of male, baseline level of proteinuria, follow-up durations, and quality score. Potential publication bias was assessed with Egger’s regression asymmetry test, or visual inspection of funnel plots if enough RCTs are included [39]. P values were two-tailed and statistical significance was set at 0.05. We used RevMan (Version 5.1; Cochrane, Oxford, UK) and Stata 12.0 software for the meta-analysis and statistical study.

## Results

### Search results

**Figure 1.**
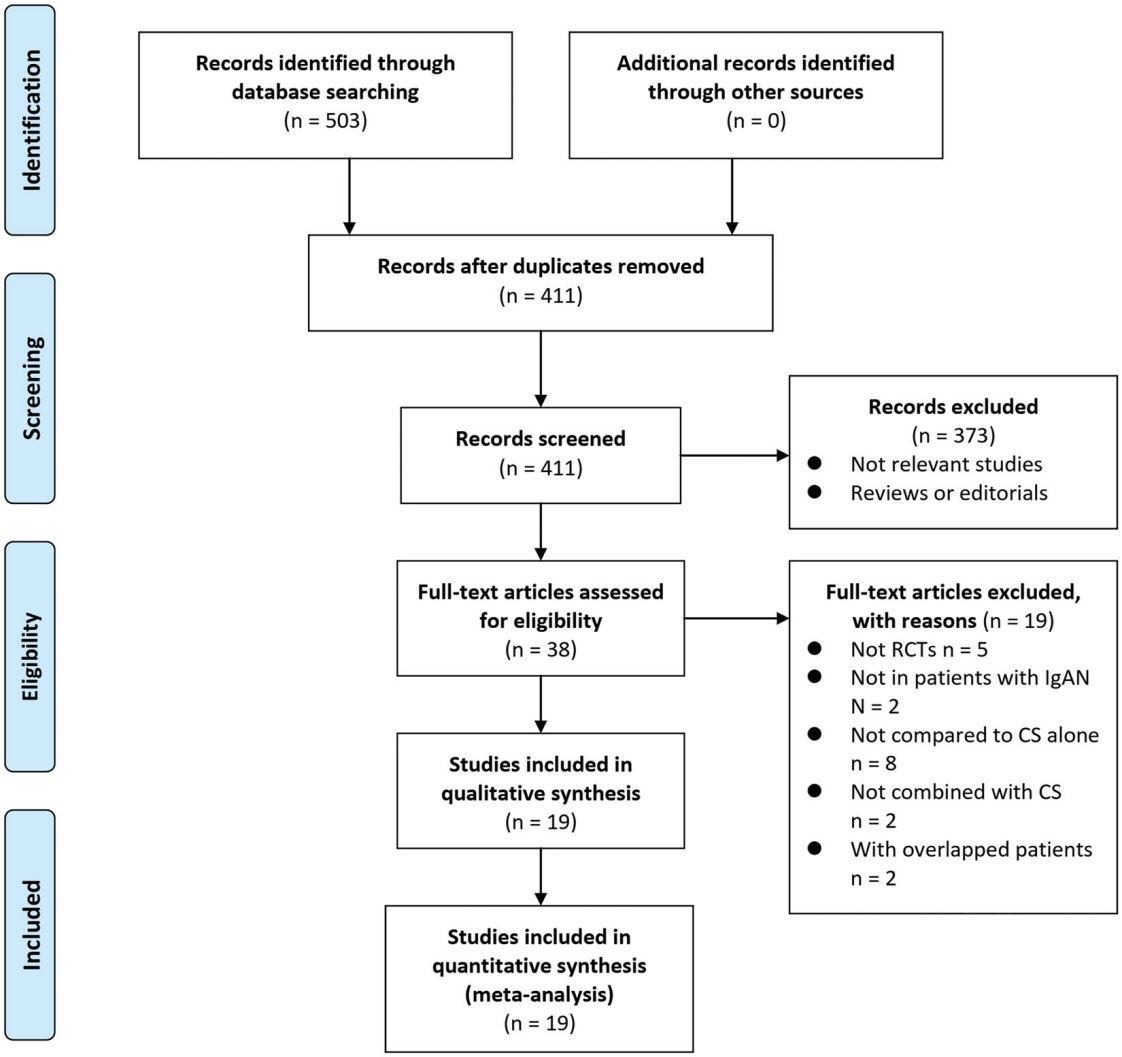
Flowchart of database search and literature identification.

A total of 503 articles were identified through database search, and 411 were retrieved after excluding the duplications. Subsequently, 373 were further excluded by screening of the titles and abstracts mainly because these studies were not relevant to the aim of the meta-analysis. Of the 38 potentially relevant articles for full-text review, nineteen studies were further excluded based on the reasons listed in Figure 1. Finally, the remaining 19 studies [16–34] met the inclusion criteria of the meta-analysis and were finally included for subsequent analyses.

### Study characteristics

Overall, nineteen RCTs [16–34] including 1153 patients with IgAN were included in the meta-analysis. The characteristics of the included studies are shown in Table 1. All of the included studies were open-label and parallel-group RCTs performed in China. All of the RCTs included patients with biopsy-proved IgAN, with the duration of the disease varying between 3 months and 10 years. The sample sizes of the studies varied between 36 and 108, and the mean ages of the patients ranged between 32 and 43 years. The proportions of males varied between 42% and 72%. The dosages of leflunomide were maintained as 20 mg/day, with or without a loading dose of 40 or 50 mg/day in the first three days of the treatment. Prednisone was used in both the interventional and control groups, with a dosage of 0.5–1 mg/kg/day and tapered thereafter. The follow-up durations varied from 1 to 24 months. Supporting treatments such as ACEIs/ARBs were generally concurrently administered. In ten studies [19,21,24,26–30,32,33], the dosages of CS was equal between the combined and the control groups (studies with full-dose CS in the combined treatment), while in nine studies [16–18,20,22,23,25,31,34], the dosages of CS used in the combined therapy was lower than that of the control group (studies with reduced dose of CS in the combined treatment).

**Table 1.** Characteristics of the included studies.

Author year	Study design	Inclusion criteria	No of patients	Male	Age	Duration of IgAN	Baseline proteinuria	Baseline SCr	Baseline eGFR	Lee Classification	Intervention (LEF + CS) regimen	Control (CS) regimen	Treatment duration	ACEI/ARB use	Definitions of CR and PR	Outcomes reported
				%	Years	Years	g/day	umol/L	mL/min	I/II/III/IV/V			months	%		
Studies with full dose of CS in the combined group																
Sun 2009 [19]	R, OL	Biopsy proven IgAN; Proteinuria 1–3.5 g/day	36	56	37	6 m	2.7	NA	NA	III–V	LEF: 50 mg/day for 3d, then reduced to 20 mg/day; P: 1 mg/kg/day, then tapered	P: 1 mg/kg/day, then tapered	3	Yes	CR: proteinuri*a* < 0.3 g/day and a normal SCr; PR: proteinuria reducin*g* ≥ 50% and SCr stable	①②③⑥⑦
Zhang 2010 b [22]	R, OL	Biopsy proven IgAN; SC*r* < 256 umol/L; Proteinuria 1–3 g/day	30	43	43	3 m–3 y	2.3	109	40	III-V	LEF: 50 mg/day for 3d, then reduced to 20 mg/day; P: 1 mg/kg/day, then tapered	P: 1 mg/kg/day, then tapered	6	None	CR: proteinuri*a* < 0.15 g/day and a normal SCr; PR: proteinuria reducin*g* ≥ 50% and SCr stable	①②③④⑤⑦
Li 2011 [24]	R, OL	Biopsy proven IgAN; Proteinuri*a* ≥ 1 g/day	60	62	37	2 y	2.5	109	NA	0/0/23/30/7	LEF: 50 mg/day for 3 days, then reduced to 20 mg/day; P: 0.5 mg/kg/day, then tapered	P: 0.5 mg/kg/day, then tapered	6	Yes	CR: proteinuri*a* < 0.2 g/day and a normal SCr; PR: proteinuria reducin*g* ≥ 50% and SCr stable	①②③④⑦
Wang 2014 [26]	R, OL	Biopsy proven IgAN; SC*r* < 130 umol/L; Proteinuria 1–3 g/day	40	50	NA	NA	2.3	70	NA	NA	LEF: 50 mg/day for 3 days, then reduced to 20 mg/day; P: 1 mg/kg/day, then tapered	P: 1 mg/kg/day, then tapered	6	NA	CR: proteinuri*a* < 0.15 g/day and a normal SCr; PR: proteinuria reducin*g* ≥ 50% and SCr stable	①②③④⑤
Wu 2014 [27]	R, OL	Biopsy proven IgAN; SC*r* < 256 umol/L; Proteinuri*a* ≥ 3.5 g/day	60	55	35	1.6 y	4.7	85	NA	0/17/32/11/0	LEF: 20 mg/day; P: 1 mg/kg/day, then tapered	P: 1 mg/kg/day, then tapered	12	NA	CR: proteinuri*a* < 0.2 g/day and a normal SCr; PR: proteinuria reducin*g* ≥ 50% and SCr stable	①②③④⑦⑨
Yang 2016 [29]	R, OL	Biopsy proven IgAN; Proteinuri*a* ≥ 1 g/day	104	56	34	8–35 m	1.9	105	78	0/52/33/19	LEF: 50 mg/day for 3 days, then reduced to 20 mg/day; P: 0.5 mg/kg/day, then tapered	P: 0.5 mg/kg/day, then tapered	6	Yes	CR: proteinuri*a* < 0.2 g/day and a normal SCr; PR: proteinuria reducin*g* ≥ 50% and SCr stable	①②③④⑤⑥⑦⑧
Li 2016 [28]	R, OL	Biopsy proven IgAN; Proteinuri*a* ≥ 3.5 g/day	80	54	38	2 y	4.2	99	NA	NA	LEF: 50 mg/day for 3 days, then reduced to 20 mg/day; P: 0.5 mg/kg/day, then tapered	P: 0.5 mg/kg/day, then tapered	3	NA	CR: proteinuri*a* < 0.2 g/day and a normal SCr; PR: proteinuria reducin*g* ≥ 50% and SCr stable	②③④⑥⑦
Lin 2017 [30]	R, OL	Biopsy proven IgAN; Proteinuri*a* ≥ 3.5 g/day	80	58	35	2 y	3.7	86	78	0/15/29/29/7	LEF: 20 mg/day; P: 1 mg/kg/day, then tapered	P: 1 mg/kg/day, then tapered	3	Yes	CR: proteinuri*a* < 0.2 g/day and a normal SCr; PR: proteinuria reducin*g* ≥ 50% and SCr stable	①②③④⑤⑥
Zheng 2020 [32]	R, OL	Biopsy proven IgAN; Proteinuri*a* ≥ 1 g/day	64	48	37	1.6 y	2.4	98	NA	0/21/26/17	LEF: 20 mg/day; P: 1 mg/kg/day, then tapered	P: 1 mg/kg/day, then tapered	6	NA	CR: proteinuri*a* < 0.3 g/day and a normal SCr; PR: proteinuria reducin*g* ≥ 50% and SCr stable	①②③④⑥⑦⑧
Zhu 2020 [33]	R, OL	Biopsy proven IgAN; eGFR: > 30 ml/min;	106	60	43	1.8 y	2.5	94	58	0/31/45/30	LEF:50mg/day for 3 days, then reduced to 20 mg/day; P: 0.5 mg/kg/day, then tapered	P: 0.5 mg/kg/day, then tapered	12	Yes	NA	③④⑤
Studies with reduced dose of CS in the combined group																
Wang 2006 [17]	R, OL	Biopsy proven IgAN; SC*r* < 355 umol/L; Proteinuri*a* ≥ 1 g/day	36	72	36	10.2 y	2.4	188	39	0/0/9/24/3	LEF: 50 mg/day for 3 days, then reduced to 20 mg/day; P: 0.5 mg/kg/day, then tapered	P: 0.8–1 mg/kg/day, then tapered	6	Yes	CR: proteinuri*a* < 0.3 g/day and a normal SCr;	①③④⑤⑥⑦
Fu 2006 [16]	R, OL	Biopsy proven IgAN; eGFR: 29–60ml/min; Proteinuri*a* ≥ 1 g/day	60	60	43	3 m	2.6	185	47	NA	LEF: 50 mg/day for 3 days, then reduced to 20 mg/day; P: 30 mg/day, then tapered	P: 1 mg/kg/day, then tapered	12	Yes	NA	①③④⑤⑦
Fu 2009 [18]	R, OL	Biopsy proven IgAN; Proteinuri*a* ≥ 1 g/day	37	51	34	10.5 m	2.5	112	NA	6/17/10/4/0	LEF: 50 mg/day for 3 days, then reduced to 20 mg/day; P: 20 mg/day, then tapered	P: 0.8–1 mg/kg/day, then tapered	1	None	CR: proteinuri*a* < 0.2 g/day and a normal SCr; PR: proteinuria reducin*g* ≥ 50% and SCr stable	①②③④⑥⑦
Shi 2010 [20]	R, OL	Biopsy proven IgAN; Proteinuri*a* ≥ 1 g/day	36	47	40	6 m	2.5	89	76	II–IV	LEF: 40 mg/day for 3 days, then reduced to 20 mg/day; P: 0.8 mg/kg/day, then tapered	P: 1 mg/kg/day, then tapered	12	Yes	CR: proteinuri*a* < 0.2 g/day and a normal SCr; PR: proteinuria reducin*g* ≥ 50% and SCr stable	①②③④⑤⑥⑦⑧⑩
Zhang 2010a [21]	R, OL	Biopsy proven IgAN; Proteinuri*a* ≥ 1 g/day	42	67	38	NA	2.9	193	41	III-V	LEF: 50 mg/day for 3 days, then reduced to 20 mg/day; P: 0.8 mg/kg/day, then tapered	P: 1 mg/kg/day, then tapered	9	Yes	CR: proteinuri*a* < 0.2 g/day and a normal SCr; PR: proteinuria reducin*g* ≥ 50% and SCr stable	①②⑥⑦
Hu 2011 [23]	R, OL	Biopsy proven IgAN; SC*r* < 256 umol/L; Proteinuria 1–3 g/day	47	66	43	NA	2.1	96	NA	5/9/22/10/1	LEF: 50 mg/day for 3 days, then reduced to 20 mg/day; P: 0.6 mg/kg/day, then tapered	P: 1 mg/kg/day, then tapered	9	Yes	CR: proteinuri*a* < 0.3 g/day and a normal SCr; PR: proteinuria reducin*g* ≥ 50% and SCr stable	①②③④⑦
Shen 2012 [25]	R, OL	Biopsy proven IgAN; Proteinuri*a* ≥ 1 g/day	42	NA	32	NA	3.1	106	NA	NA	LEF: 50 mg/day for 3 days, then reduced to 20 mg/day; P: 24 mg/day, then tapered	P: 32–40 mg/day, then tapered	6	NA	CR: proteinuri*a* < 0.4 g/day and a normal SCr; PR: proteinuria reducin*g* ≥ 50% and SCr stable	①②③④⑥⑦⑧⑨⑩
Min 2017 [31]	R, OL	Biopsy proven IgAN; eGFR: > 30 mL/min; Proteinuri*a* ≥ 1 g/day	85	42	37	NA	1.8	94	84	NA	LEF: 40 mg/day for 3 days, then reduced to 20 mg/day; P: 0.8 mg/kg/day, then tapered	P: 1 mg/kg/day, then tapered	12	Yes	CR: proteinuri*a* < 0.3 g/day and a normal SCr; PR: proteinuria reducin*g* ≥ 50% and SCr stable	①②③④⑤⑥⑦⑧⑨
Ni 2021 [34]	R, OL	Biopsy proven IgAN; eGFR: 30–60 mL/min; Proteinuri*a* ≥ 1 g/day	108	51	36	1.3 y	1.9	98	83	NA	LEF:50mg/day for 3 days, then reduced to 20 mg/day; P: 0.5–0.8 mg/kg/day, then tapered	P: 1 mg/kg/day, then tapered	24	Yes	CR: proteinuri*a* < 0.3 g/day and a normal SCr; PR: proteinuria reducin*g* ≥ 50% and SCr stable	①②③④⑤⑥⑦⑧⑩

Outcomes reported.

^①^, CR; ^②^, CR + PR; ^③^, UPE; ^④^, SCr; ^⑤^, eGFR; ^⑥^, GI discomfort; ^⑦^, elevated ALT/AST; ^⑧^, infection; ^⑨^, elevated glucose; ^⑩^ elevated BP;.

R: randomized controlled trials; OL: open-label; IgAN: immunoglobulin A nephropathy; SCr: serum creatinine; eGFR: estimated glomerular filtration rate; LEF: leflunomide; CS: corticosteroid; P: prednisone; ACEI: angiotensin converting enzyme inhibitor; ARB: angiotensin II receptor blockers; CR: complete remission; PR: partial remission; NA: not available; m: month; y: year; UPE: urine protein excretion; GI: gastrointestinal; ALT: alanine aminotransferase; AST: aspartate aminotransferase; BP: blood pressure.

### Data quality

The details of risks of biases of the included studies according to the Cochrane assessment tool are listed in Table 2. The details of random sequence generation were reported in five studies [28–30,32,34], and the details allocation concealment were reported in two studies [20,34]. The details of withdrawals and dropouts were reported in all studies.

**Table 2.** Quality evaluation *via* the Cochrane’s risk of bias tool.

	Random sequence generation	Allocation concealment	Blinding in performance	Blinding in outcome detection	Incomplete outcome data	Reporting bias	Other bias	Total
Wang 2006 [17]	Unclear	Unclear	High	High	Low	Low	Low	3
Fu 2006 [16]	Unclear	Unclear	High	High	Low	Low	Low	3
Fu 2009 [18]	Unclear	Unclear	High	High	Low	Low	Low	3
Sun 2009 [19]	Unclear	Unclear	High	High	Low	Low	Low	3
Shi 2010 [20]	Unclear	Low	High	High	Low	Low	Low	4
Zhang 2010a [22]	Unclear	Unclear	High	High	Low	Low	Low	3
Zhang 2010b [21]	Unclear	Unclear	High	High	Low	Low	Low	3
Li 2011 [24]	Unclear	Unclear	High	High	Low	Low	Low	3
Hu 2011 [23]	Low	Unclear	High	High	Low	Low	Low	4
Shen 2012 [25]	Unclear	Unclear	High	High	Low	Low	Low	3
Wang 2014 [26]	Unclear	Unclear	High	High	Low	Low	Low	3
Wu 2014 [27]	Unclear	Unclear	High	High	Low	Low	Low	3
Yang 2016 [29]	Low	Unclear	High	High	Low	Low	Low	4
Li 2016 [28]	Low	Unclear	High	High	Low	Low	Low	4
Lin 2017 [30]	Low	Unclear	High	High	Low	Low	Low	4
Min 2017 [31]	Unclear	Unclear	High	High	Low	Low	Low	3
Zheng 2020 [32]	Low	Unclear	High	High	Low	Low	Low	4
Zhu 2020 [33]	Unclear	Unclear	High	High	Low	Low	Low	3
Ni 2021 [34]	Low	Low	High	Low	Low	Low	Low	6

### Efficacy outcomes

**Figure 2.**
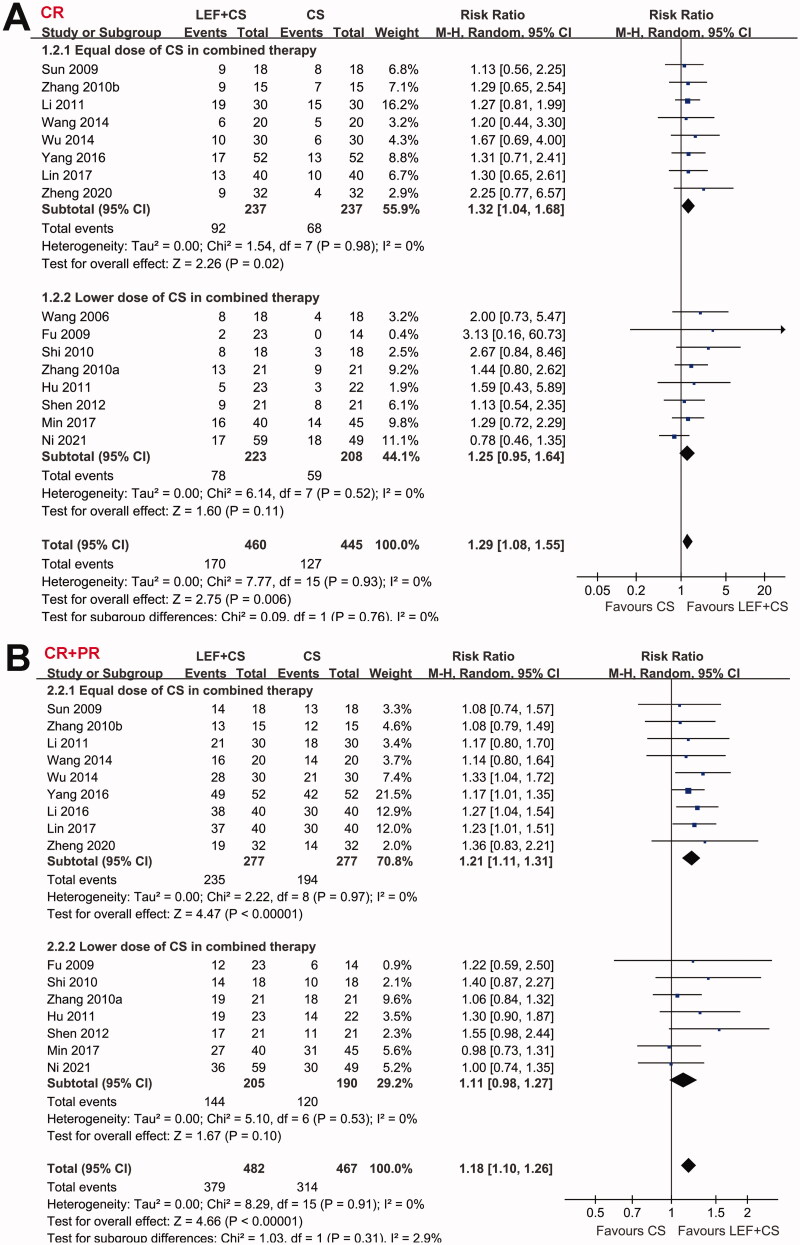
Forest plots for the meta-analyses comparing the combined therapy with leflunomide and CS versus control group of CS alone on the CR and overall response of proteinuria in patients with IgAN; A, forest plots for the incidence of CR of proteinuria; and B, forest plots for the overall response.

**Figure 3.**
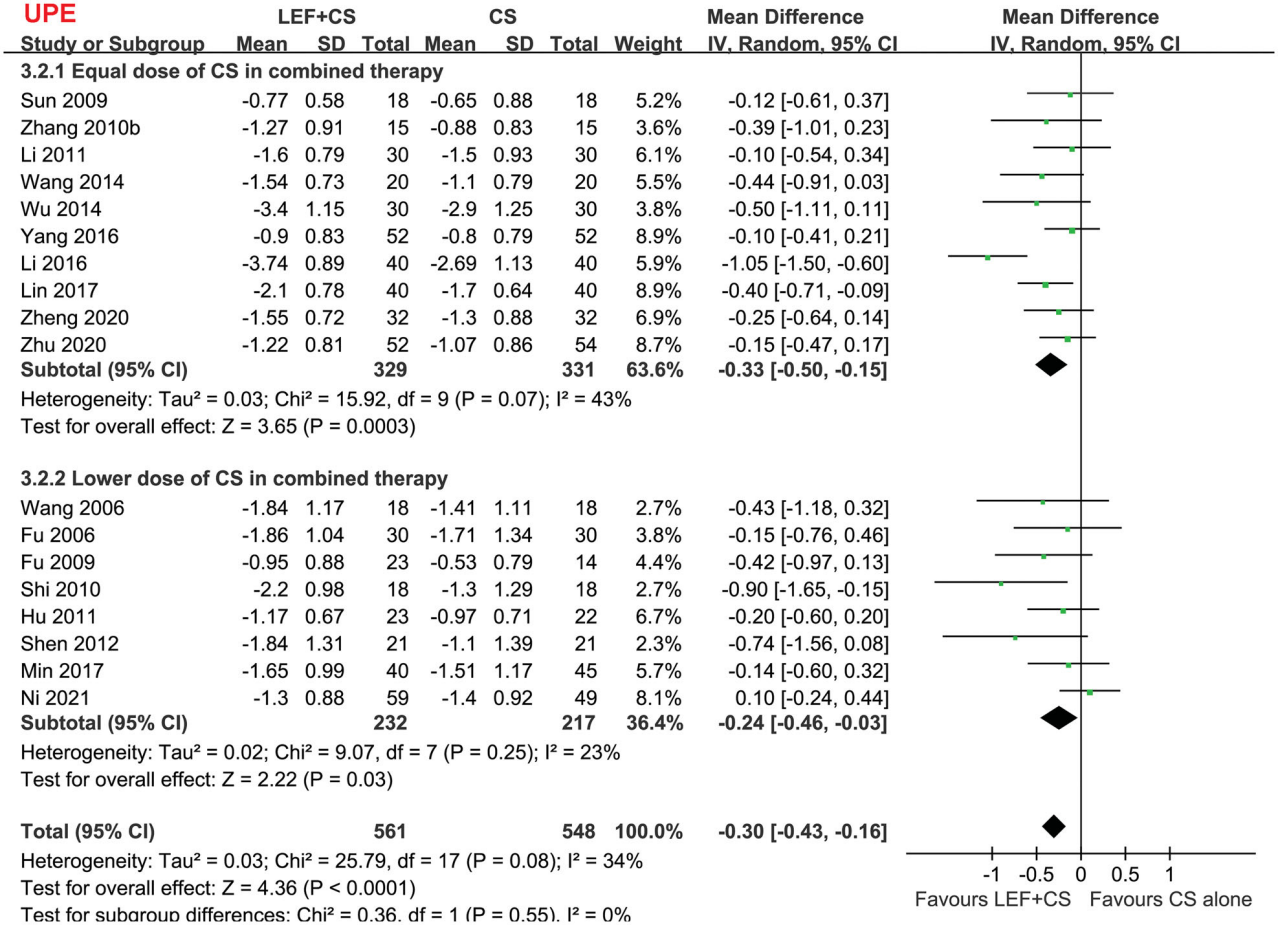
Forest plots for the meta-analyses comparing the combined therapy with leflunomide and CS versus control group of CS alone on UPE in patients with IgAN.

**Figure 4.**
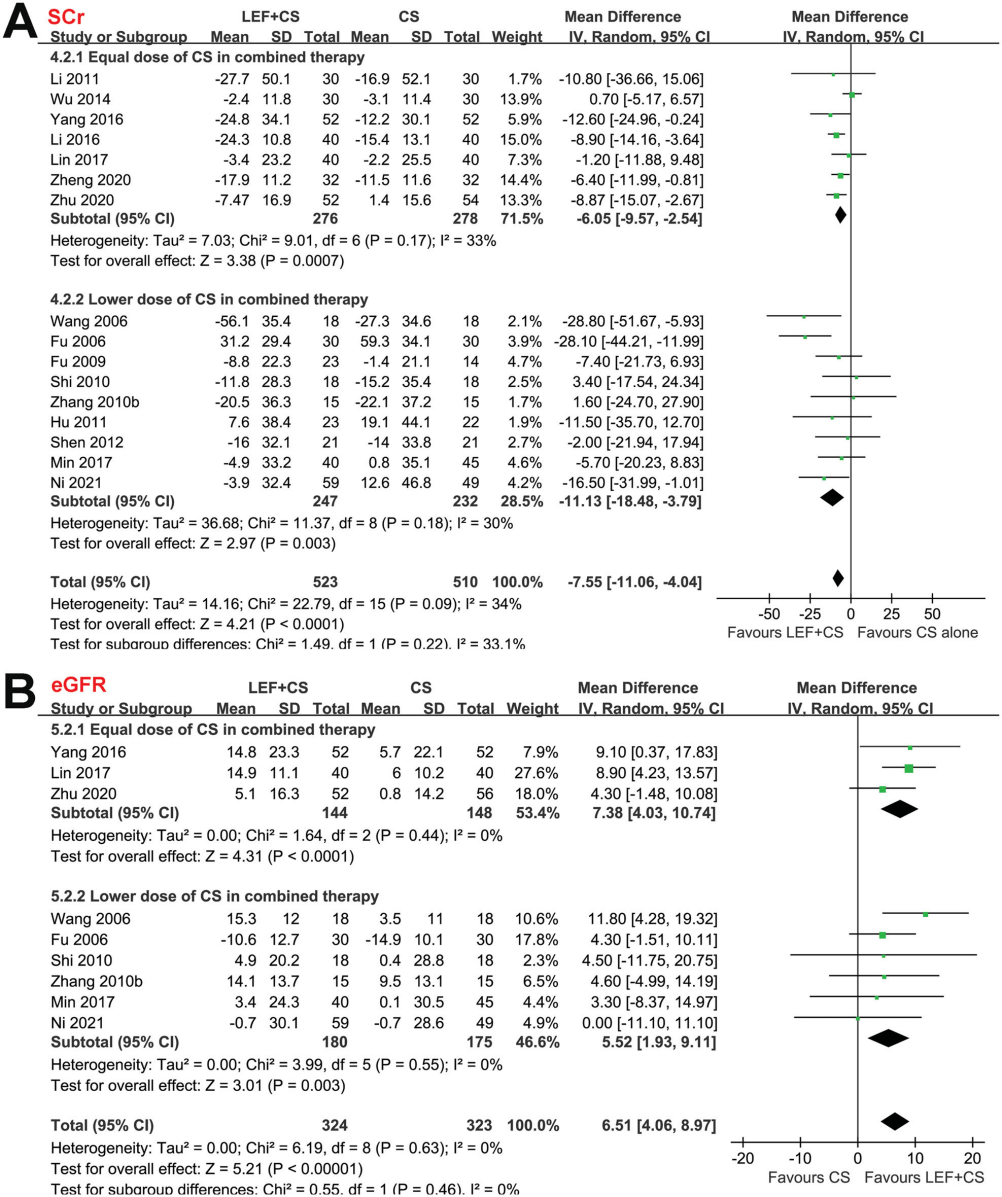
Forest plots for the meta-analyses comparing the combined therapy with leflunomide and CS versus control group of CS alone on renal function in patients with IgAN; (A) forest plots for the changes of SCr; and (B) forest plots for the changes of eGFR.

Pooled results showed that patients who received combined therapy had a higher rate of CR compared to patients who received CS alone (RR: 1.29, 95% CI: 1.08–1.55, *p* = 0.006; Figure 2(A)) with no significant heterogeneity (P for Cochrane’s Q test = 0.93, *I*^2^ = 0%). Subgroup analyses showed consistent results in full-dose studies (RR: 1.32, 95% CI: 1.04–1.68, *p* = 0.02, *I*^2^ = 0%), but the effect was not significant in studies with reduced dose of CS in the combined treatment (RR: 1.25, 95% CI: 0.95–1.64, *p* = 0.11, *I*^2^ = 0%). Moreover, the overall response rate, defined as CR and PR of proteinuria, was also significantly higher in patients allocated to the combined therapy (RR: 1.18, 95% CI: 1.10–1.26, *p* < 0.001, *I*^2^ = 0%; Figure 2(B)). Subgroup analyses also showed consistent results in full-dose studies (RR: 1.21, 95% CI: 1.11–1.31, *p* < 0.001, *I*^2^ = 0%), but the effect was not significant in studies with reduced dose of CS in the combined treatment (RR: 1.11, 95% CI: 0.98–1.27, *p* = 0.10, *I*^2^ = 0%). In addition, combined therapy of leflunomide and CS was associated with significantly reduced UPE (MD: −0.30 g/24h, 95% CI: −0.43 to −0.16, *p* < 0.001; *I*^2^ = 34%; Figure 3) and SCr (MD: −7.55 mmol/L, 95% CI: −11.06 to −4.04, *p* < 0.001; *I*^2^ = 34%; Figure 4(A)), and an increased EGFR (MD: 6.51 mL/min/1.73m^2^, 95% CI: 4.06 to 8.97, *p* < 0.001; *I*^2^ = 0%; Figure 4(B)). Subgroup analyses showed similar results in studies with full-dose or reduced dose of CS in the combined treatment (all subgroup effects < 0.05 for the outcomes of UPE, SCr, and eGFR). For the efficacy outcomes including CR, overall response rate, UPE, and SCr, results of meta-regression analyses showed that study characteristics such as sample size, mean age, proportion of male, proteinuria at baseline, follow-up duration, or quality score did not significantly modify the efficacy of the combined therapy with leflunomide and CS for patients with IgAN (*p* all > 0.05; Table 3).

**Table 3.** Results of univariate meta-regression analysis.

Covariate	Coefficient	95% CI	*p*
CR of proteinuria			
No. of patients	−0.012	−0.039 to 0.015	0.49
Mean age (years)	−0.051	−0.131 to 0.029	0.20
Male (%)	0.016	−0.017 to 0.049	0.63
Proteinuria at baseline (g/day)	0.091	−0.018 to 0.200	0.17
Follow-up duration (months)	0.072	−0.054 to 0.198	0.35
Quality score	0.130	−0.080 to 0.341	0.16
Overall response			
No. of patients	0.020	−0.048 to 0.088	0.55
Mean age (years)	−0.037	−0.093 to 0.019	0.34
Male (%)	0.033	−0.010 to 0.076	0.18
Proteinuria at baseline (g/day)	−0.032	−0.095 to 0.031	0.13
Follow-up duration (months)	0.051	−0.050 to 0.151	0.36
Quality score	0.208	−0.521 to 0.937	0.80
UPE (g/24h)			
No. of patients	−0.312	−0.845 to 0.221	0.52
Mean age (years)	−0.211	−0.505 to 0.083	0.20
Male (%)	0.052	−0.044 to 0.148	0.31
Proteinuria at baseline (g/day)	−0.016	−0.049 to 0.017	0.63
Follow-up duration (months)	0.037	−0.019 to 0.093	0.18
Quality score	−0.320	−0.752 to 0.112	0.25
SCr(mmol/L)			
No. of patients	−0.157	−0.352 to 0.038	0.13
Mean age (years)	−0.130	−0.405 to 0.145	0.42
Male (%)	−0.118	−0.244 to 0.008	0.12
Proteinuria at baseline (g/day)	−0.052	−0.141 to 0.037	0.26
Follow-up duration (months)	0.173	−0.050 to 0.396	0.19
Quality score	−0.288	−0.796 to 0.220	0.38

CR: complete remission; CI: confidence interval; UPE: urine protein excretion; SCr: serum creatinine.

## Safety outcomes

**Figure 5.**
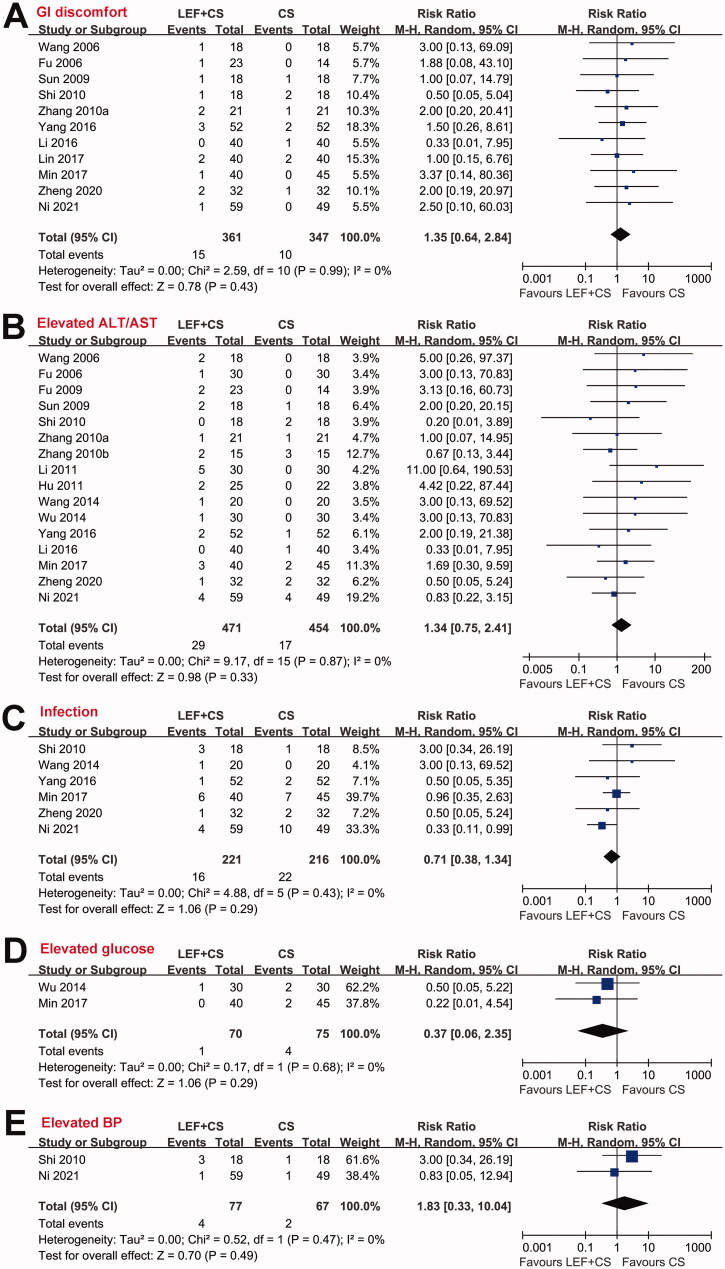
Forest plots for the meta-analyses comparing the incidence of adverse events between patients treated with a combined therapy and CS alone; (A) incidence of any GI discomfort; (B) incidence of elevated ALT/AST; (C) incidence of infection; (D) incidence of elevated glucose that required medical treatment; and (E) incidence of elevated BP that required medical treatment.

Pooled results showed that the incidence of adverse events were similar between the two groups, including GI discomfort (RR: 1.35, 95% CI: 0.64–2.84, *p* = 0.43, *I*^2^ = 0%; Figure 5(A)), elevated ALT/AST (RR: 1.34, 95% CI: 0.75–2.41, *p* = 0.33, *I*^2^ = 0%; Figure 5(B)), infection (RR: 0.71, 95% CI: 0.38–1.34, *p* = 0.29, *I*^2^ = 0%; Figure 5(C)), elevated glucose (RR: 0.37, 95% CI: 0.06–2.35, *p* = 0.29, *I*^2^ = 0%; Figure 5(D)), and elevated BP (RR: 1.83, 95% CI: 0.33 to 10.04, *p* = 0.49, *I*^2^ = 0%; Figure 5(E)) that require medical treatments.

### Publication bias

**Figure 6.**
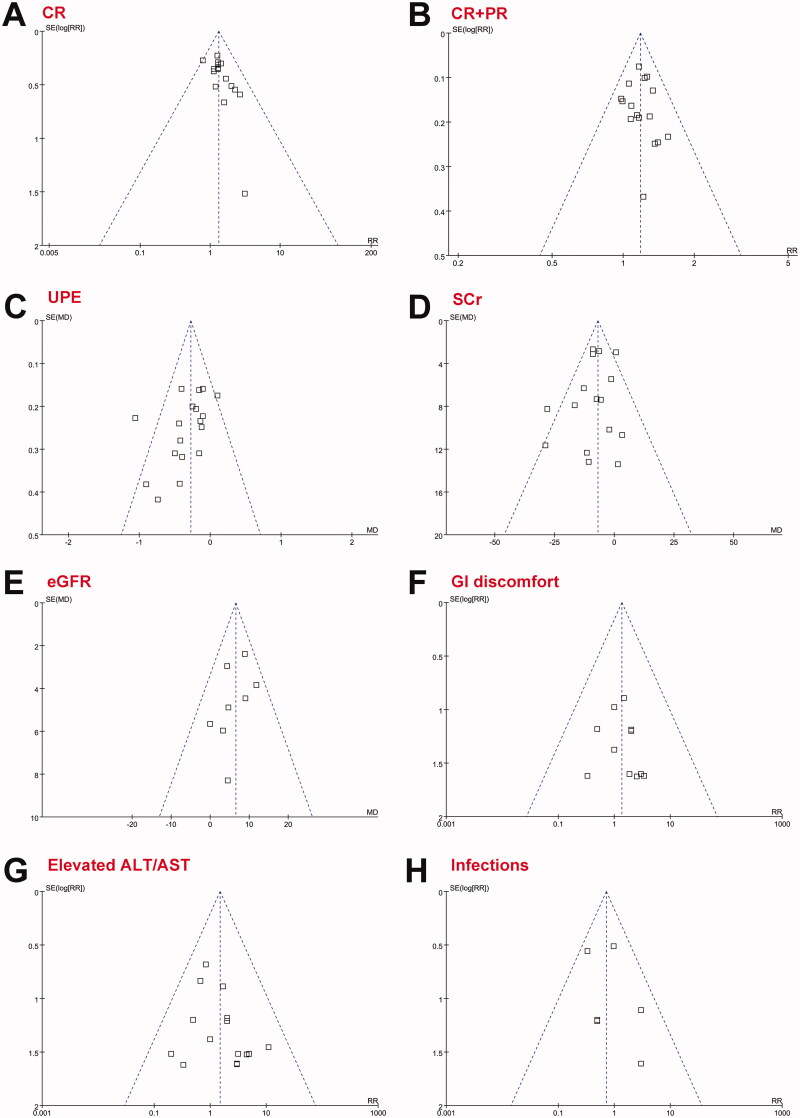
Funnel plot for the evaluation of publication biases of the meta-analyses; (A) CR of proteinuria; (B) overall response; (C) changes of UPE; (D) changes of SCr; (E) changes of eGFR; F) incidence of any GI discomfort; (G) incidence of elevated ALT/AST; and (H) incidence of infection. The full search strategy for PubMed. (‘IgA nephropathy’ OR ‘immunoglobulin A nephropathy’ OR ‘IgA nephritis’ OR ‘IgA glomerulonephritis’ OR ‘Berger's disease’ OR ‘IgAN’) AND (leflunomide) AND (random OR randomly OR randomized OR randomized OR placebo OR controlled).

Forest plots for the meta-analyses comparing the combined treatment and CS alone on outcomes including CR of proteinuria, overall response, UPE, SCr, eGFR, and adverse events such as GI discomfort, elevated ALT/AST, and infection were shown in Figures 6(A–H). The plots were symmetrical on visual inspection, suggesting low risks of publication biases. The results of Egger’s regression tests also suggested low risks of publication biases (P for Egger’s regression tests all > 0.05). Potential publication biases of meta-analyses of the other two outcomes (elevated glucose and elevated BP) were unable to determine because only two datasets were included.

## Discussion

Results of the meta-analysis showed that the combined treatment with leflunomide and CS significantly improved CR and overall response rate of proteinuria in patients with IgAN compared to a treatment with CS alone. These results were further validated by the reduced UPE in patients allocated to the combined treatment as compared to the patients who received CS alone. Besides, the combined treatment with leflunomide and CS is also associated with a significantly preserved renal function, as evidenced by the reduced SCr and preserved eGFR in these patients. For the rates of CR and overall response, subgroup analyses showed consistent results in full-dose studies, but the effects were not significant in studies with reduced dose of CS in the combined treatment. However, the between-subgroup difference was not significant. Further subgroup analyses of other outcomes, consistent results were obtained in studies with lower dose and equal dose of CS in the combined group. As for the safety outcome, no significant difference was detected for the incidence of adverse events, including GI discomfort, elevated ALT/AST, infection, and elevated glucose and BP that require medical treatments. Taken together, the combined treatment with leflunomide and CS is more effective than CS alone for reducing proteinuria and preserving renal function in patients with IgAN. Moreover, the combined treatment is not associated with an increased risk of adverse events. These findings support that the combined treatment with leflunomide and CS could be applied as an alternative treatment strategy for patients with IgAN.

A previous meta-analysis has evaluated the possible role of leflunomide in patients with IgAN [40]. This meta-analysis included studies published before 2019 and the results suggested possible benefits of leflunomide in the improvement of renal function and reduction of urinary protein loss in patients with IgAN [40]. However, besides of RCTs, non-randomized studies were also included in the meta-analysis, which may confound the results. Moreover, the inclusion criteria for the meta-analysis are not restricted, and studies with various treatment regimens in control groups were included, such as those with CS alone, ACEI/ARB alone, placebo or blank treatment, or the other immunosuppressants, which made the interpretation of the result difficult. Recently, a well-designed network meta-analysis was performed to evaluate the efficacy and safety of different immunosuppressants for high-risk IgAN [41]. Although this study could provide more information and consistently showed that combined leflunomide with CS could serve as one of the best choices for patients with high-risk IgAN [41], results of network meta-analysis were generally based on studies of indirect comparison, which may limit the reliability of the findings. For the comparison of LEF + CS vs CS, RCTs are adequate for a meta-analysis of head-to-head comparison, like ours. Besides, we have further performed subgroup according to whether LEF was added to full-dose or reduced dose of CS, this could not be done in a network meta-analysis. In our study, we focused our objective to compare the efficacy and safety of comparing the combined treatment with leflunomide and CS with CS alone. The results confirmed the efficacies of the combined treatment in reducing proteinuria and preserving renal function for patients with IgAN. Predefined subgroup analyses further showed consistent efficacies of the combined treatment in studies with full-dose and reduced dose of CS in the combined treatment. Previous studies showed that IgAN patients with minimal or no proteinuria were likely to have excellent long-term prognosis, and few patients would progress to ESRD within a follow-up duration of up to 9 years [42,43]. Moreover, for IgAN patients with significant proteinuria, early remission of proteinuria was associated with a significantly reduced long-term incidence of ESRD [44,45]. These findings highlighted the importance of proteinuria remission as a validated surrogate outcome for patients with IgAN. Our results found that compared to CS alone, combined treatment with leflunomide and CS further improved the CR and overall response rates of proteinuria in patients with IgAN, suggesting the possible long-term clinical benefits of the combined therapy in these patients.

The possible mechanisms underlying the therapeutic efficacy of leflunomide for IgAN may be multifactorial. As a novel immunosuppressant, leflunomide was shown to inhibit the activity of tyrosine kinases and NF-κB in T lymphocytes [46]. Besides, leflunomide was also shown to inhibit the activity of dihydrofolate dehydrogenase and cell cycle-dependent kinases, further preventing the proliferation of T and B lymphocytes and immune responses [46]. In view of the importance of autoimmunity in the pathogenesis of IgAN, leflunomide could also confer its therapeutic efficacy for IgAN as an immunosuppressant [47]. In addition, some studies also suggested a possible direct benefit of leflunomide on glomerular disease. For example, in a preclinical study of glomerulonephritis induced by the anti-basement membrane antibody, treatment with leflunomide was associated with alleviated glomerular lesions and reduced deposits of rat IgG and C3 along the glomerular capillary wall [48]. Besides, leflunomide administration was associated with improved viability and podocyte cytoskeleton in human glomerular podocytes cultured in high glucose condition [49,50]. Since injury of podocytes has been well acknowledged as an important mechanism underlying the pathogenesis of IgAN [51], the therapeutic efficacy of leflunomide for IgAN may also involve the protection of podocytes. Future studies are needed to determine the molecular pathways underlying the therapeutic efficacy of leflunomide for IgAN and the possible synergetic effect between leflunomide and CS.

Our study also has limitations. Firstly, the sample size of the included studies was limited, and the pooled results of the meta-analysis should be validated in large-scale clinical studies. Besides, the follow-up durations of the included studies were relatively short. Accordingly, this meta-analysis was not statistically adequate to investigate the possible benefit of the combined treatment on clinical outcomes such as the long-term incidence of ESRD. Moreover, all of the included studies were from China which may limit the generalizability of the study findings. Clinical trials should be performed in other countries to validate the efficacy of combined leflunomide and CS for patients with IgAN. Finally, the Oxford classification has been confirmed as an important determinant for the treatment efficacy of immunosuppressants in patients with IgAN. It remains unknown whether the benefits of the combined treatment are similar in patients with IgAN of different Oxford classifications. Future studies are also warranted.

## Conclusions

In conclusion, results of our meta-analysis showed that the combined treatment with leflunomide and CS was more effective than CS alone in reducing proteinuria and preserving renal function in patients with IgAN, without further increasing the risk of possible adverse events. These findings support that the combined treatment with leflunomide and CS is superior to CS alone for patients with IgAN.

## Supplementary Material

Supplemental Material
