# Supplementary material for: Efficacy and safety of leflunomide combined with corticosteroids for the treatment of IgA nephropathy: a Meta-analysis of randomized controlled trials
Source: Ren Fail. 2022 Jul 4;44(1):1011–25. doi: 10.1080/0886022X.2022.2085576 (PMC9262374; doi:10.1080/0886022X.2022.2085576)
Supplement: Supplemental Material [file IRNF_A_2085576_SM6792.pdf]

The full search strategy for PubMed

("IgA nephropathy" OR "immunoglobulin A nephropathy" OR "IgA nephritis" OR "IgA glomerulonephritis" OR "Berger's disease" OR "IgAN") AND (leflunomide) AND (random OR randomly OR randomized OR randomised OR placebo OR controlled)
